# Recurrent gastric intramural pseudocyst: A case report and comprehensive literature review of reported cases

**DOI:** 10.1016/j.radcr.2024.08.041

**Published:** 2024-09-03

**Authors:** Amirhossein Soltani, Mohsen Salimi, Maryam Nemati, Ali Mirshamsi

**Affiliations:** a Department of Radiology, Shiraz University of Medical Sciences, Shiraz, Iran; b Student Research Committee, School of Medicine, Shiraz University of Medical Sciences, Shiraz, Iran; c Medical Imaging Research Center, Shiraz University of Medical Sciences, Shiraz, Iran

**Keywords:** Intramural gastric pseudocyst, Pancreatic pseudocyst, Pseudocyst, Chronic pancreatitis, Necrotizing pancreatitis

## Abstract

Intramural gastric pseudocysts are extremely rare and are often associated with pancreatitis and pancreatic pseudocysts; they can lead to complex clinical presentations requiring careful diagnosis and management. We present a case of a 57-year-old man with a history of pancreatitis and pancreatic pseudocysts who was diagnosed with intramural gastric pseudocysts. The patient was diagnosed with multiple gastric intramural pseudocysts at different locations during separate admissions and imaging studies. This indicates a recurrence of gastric intramural pseudocysts. In these cases, studies rarely discuss recurrence and its underlying causes. This highlights a significant gap in the existing literature.

To provide a broader understanding, we reviewed the literature by searching major databases (PubMed, Scopus, and Web of Science) and then extracted and analyzed data from 18 articles, reaching 24 similar cases. Of the 25 patients studied (including our case), 92% were male and 8% were female. Cases had a mean age of 47.68 ± 14.82 years. Additionally, 84% of the patients had a history of alcohol consumption, and 88% had a positive history of pancreatitis. Common symptoms were abdominal pain (especially in the epigastric region), vomiting, nausea, and weight loss. In conclusion, results showed that intramural gastric pseudocysts generally occur in middle-aged men with a history of chronic or heavy alcohol consumption and pancreatitis.

## Introduction

Gastrointestinal intramural pseudocysts are extremely rare. Potentially, they may develop in the esophagus, stomach, duodenum, and colon. Among these, gastric intramural pseudocysts are very uncommon and have only been documented in case studies in the current literature up until now [1,2].

The term “intramural gastric pseudocyst” was first used by H. M. Radke and J. W. Bell in 1966. They described it as an amylase-containing fluid collection within the layers of the gastric wall, occurring after chronic pancreatitis [3]. However, today we know that it can occur after both chronic and acute pancreatitis [4].

The pathophysiology of intramural gastric pseudocysts has not been thoroughly clarified. Possible explanations are the rupturing of a pancreatic pseudocyst into the gastric walls, the existence of a fistula connecting the pancreas and the gastrointestinal tract, and the inflammation of heterotopic pancreatic tissue located in the gastrointestinal tract wall [1,5].

In this case report, we will describe a patient with recurrent intramural gastric cysts following chronic pancreatitis. Studies have rarely discussed this condition in terms of recurrence, its underlying causes, and pathophysiology, which highlights a significant gap in the existing literature. Then we will look into the imaging characteristics of this condition and review the literature to gather reported cases. Finally, we will perform a comprehensive review of the literature and an overview of reported cases to go much deeper into the disease and to guide future research and clinical management of this condition by addressing the current gap in the literature.

## Case presentation

A 57-year-old man with a medical history of diabetes mellitus, chronic pancreatitis, chronic smoking, and ischemic heart disease and no history of alcohol consumption, was diagnosed with intramural gastric pseudocyst. Over the past 4 years, the patient has multiple hospitalizations with similar symptoms.

**Fig. 1:**
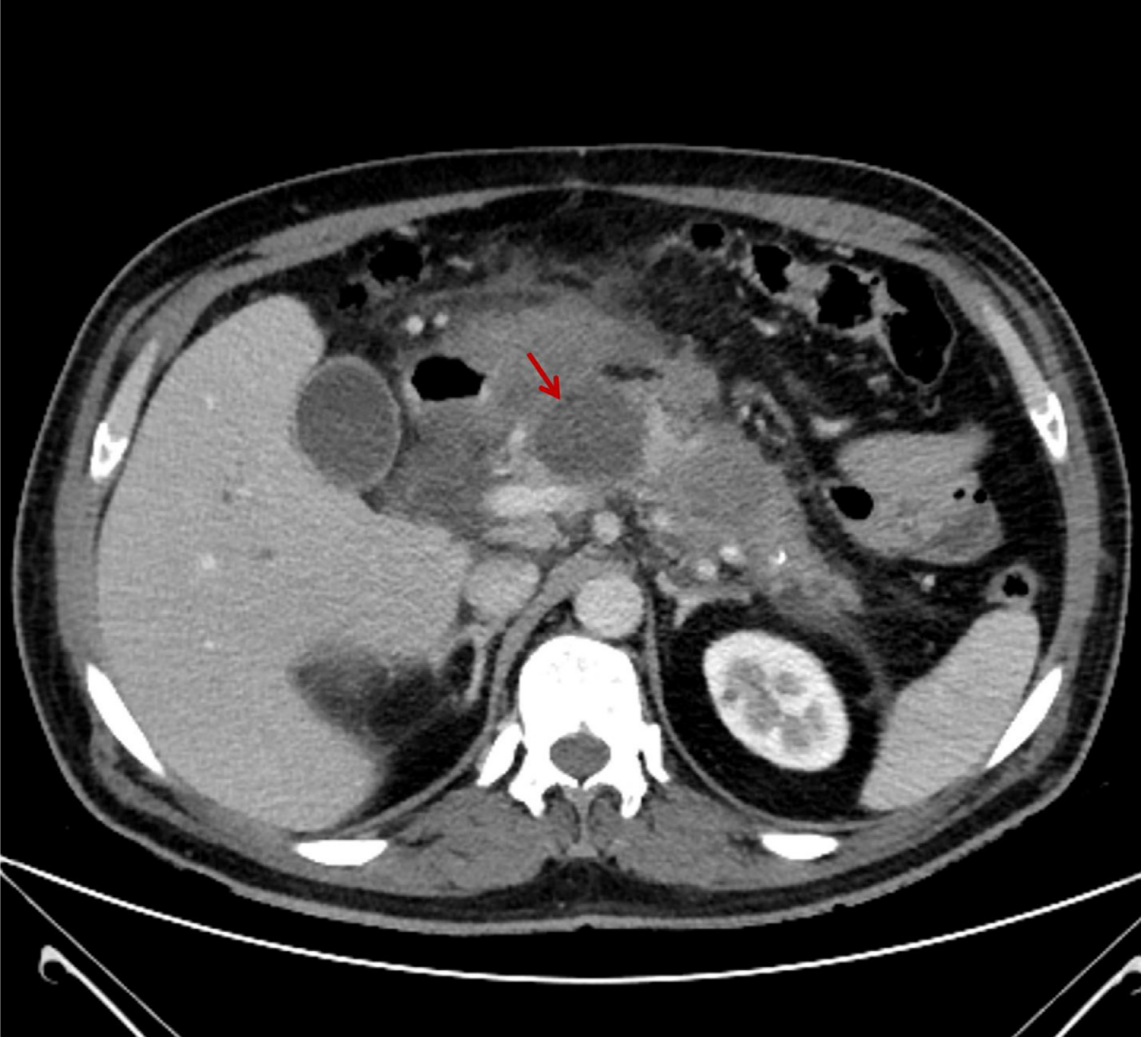
Abdominal CT scan–Axial view: The pancreas shows diffuse inhomogeneous parenchymal density and enhancement. Some areas of decreased enhancement are seen in the pancreatic tail, associated with peri-pancreatic fat stranding, suggestive of acute necrotizing pancreatitis. Evidence of pseudocyst formation is noted in the pancreas, measuring 38 × 43 mm (red arrow).

During his first hospitalization 4 years ago (first hospitalization), the patient was diagnosed with acute necrotizing pancreatitis complicated by multiple pancreatic pseudocysts (Fig. 1). The pseudocysts caused a compression effect on the gastric outlet, leading to gastric outlet obstruction. Left portal vein thrombosis was also noted in a series of patient imaging studies. The patient underwent an exploratory laparotomy. A cholecystectomy was performed due to cholecystitis with stones, and after that, drainage of the pancreatic pseudocysts was done with partial resection of the body of the pancreas.

**Fig. 2:**
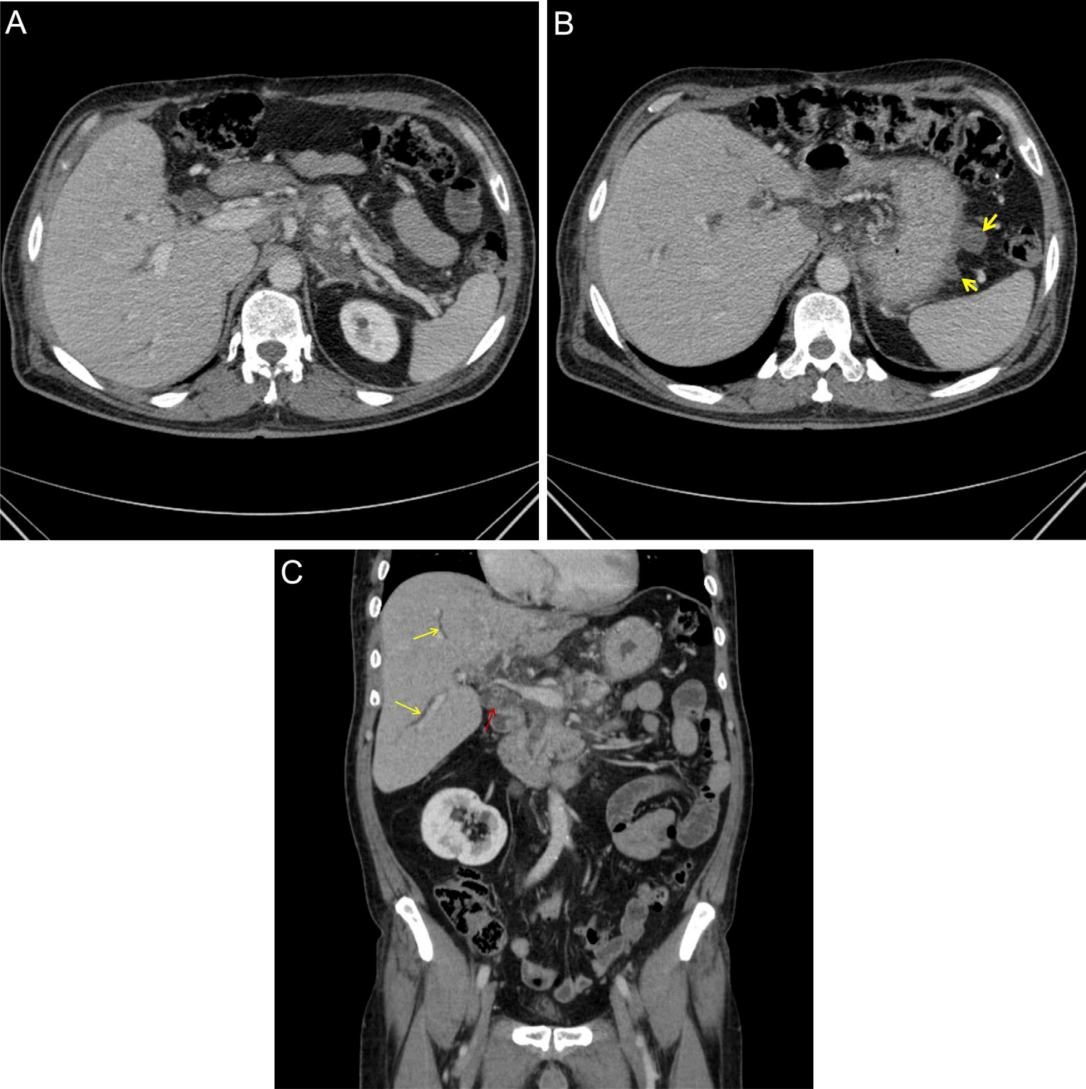
(A-C) Abdomen and pelvic CT scan, Axial and Coronal views: (A) The head and tail of the pancreas show normal size with mild inhomogeneous parenchymal density. The body of the pancreas is not seen due to previous resection. No evidence of pancreatic pseudocyst is seen. (B) Evidence of 2 newly cystic structures measuring up to 20 × 19 mm is seen in the greater curvature of the stomach. According to the patient's medical history, this is suggestive of an intramural gastric pseudocyst (2 yellow arrows). (C) Multiple stones are seen in the common bile duct (CBD) (red arrow), associated with dilatation of the common bile duct (red arrow) and intrahepatic bile ducts (yellow arrows) as the cause of recurrent pancreatitis.

The patient returned to the hospital again after 2 years (second hospitalization), showing symptoms that were similar to the previous ones. He has been diagnosed with pancreatitis once again. An abdominal CT scan revealed the existence of 2 new cystic structures located in the greater curvature of the fundus and stomach body (Fig. 2). The patient was discharged after receiving conservative treatment.

A year ago, a patient was admitted due to a urinary tract infection (UTI) (third hospitalization), in his abdominal CT scan, we observed several peripheral enhancing cystic structures in the upper part of the stomach, which were associated with significant adjacent mesenteric fat inflammation. The gastro-esophageal junction revealed the largest cystic structure, along with the presence of lymphadenopathy. These findings were consistent with intramural gastric pseudocysts. However, due to his being asymptomatic, no further workup was done for him.

In this last and present admission (fourth and present hospitalization), the patient presented with epigastric pain, nausea, and postprandial vomiting (nonbloody, nonbilious) since a day ago. He was hemodynamically stable with a normal temperature. On physical examination, he had mild tenderness in the epigastric region without any abdominal distention. The patient's blood tests showed a high total leukocyte count (WBC) of 13.8×10^3/µL (normal reference range: 4.8-10.8×10^3/µL), elevated C-reactive protein (CRP) of 66 mg/L (normal reference range: <6 mg/L), erythrocyte sedimentation rate (ESR) of 20 mm/h (normal reference range: <20 mm/h), elevated serum amylase level of 558 IU/L (normal reference range: 20-100 IU/L), elevated serum lipase of 608 IU/L (normal reference range: 5-60 IU/L), and elevated serum alkaline phosphatase (ALP) of 866 IU/L (normal reference range: 100-390 IU/L). The rest of the liver function tests were unremarkable.

**Fig. 3:**
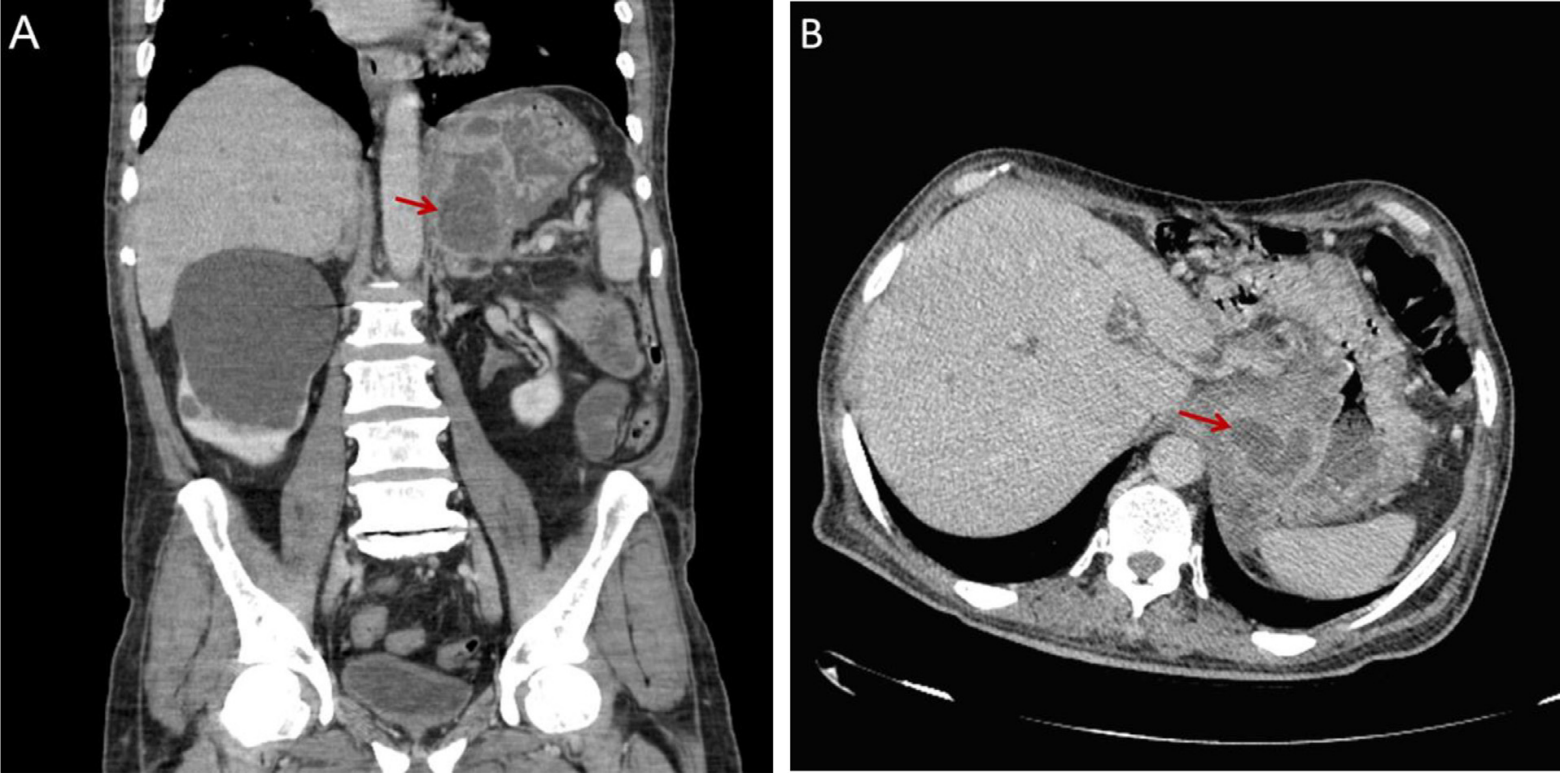
(A and B) Abdomen and pelvic CT scan, coronal and axial Views: A newly relatively thick-walled cystic structure measuring 65 × 53 mm is seen in the epigastric region, arising from the lesser curvature of the stomach, suggestive of an intramural gastric pseudocyst (red arrow). This is in addition to the 2 previously noted cystic structures in the greater curvature.

The computed tomography (CT scan) revealed evidence of multiple cystic structures of varying sizes with peripheral enhancement seen in both the lesser and greater curvatures of the stomach, suggestive of gastric intramural pseudocysts. In comparison with previous studies, a new cystic structure was observed, indicating recurrent gastric intramural pseudocysts in the patient (Fig. 3). Due to dilation of the common bile duct and intrahepatic bile ducts, performing magnetic resonance cholangiopancreatography (MRCP) was suggested for further correlation.

**Fig. 4:**
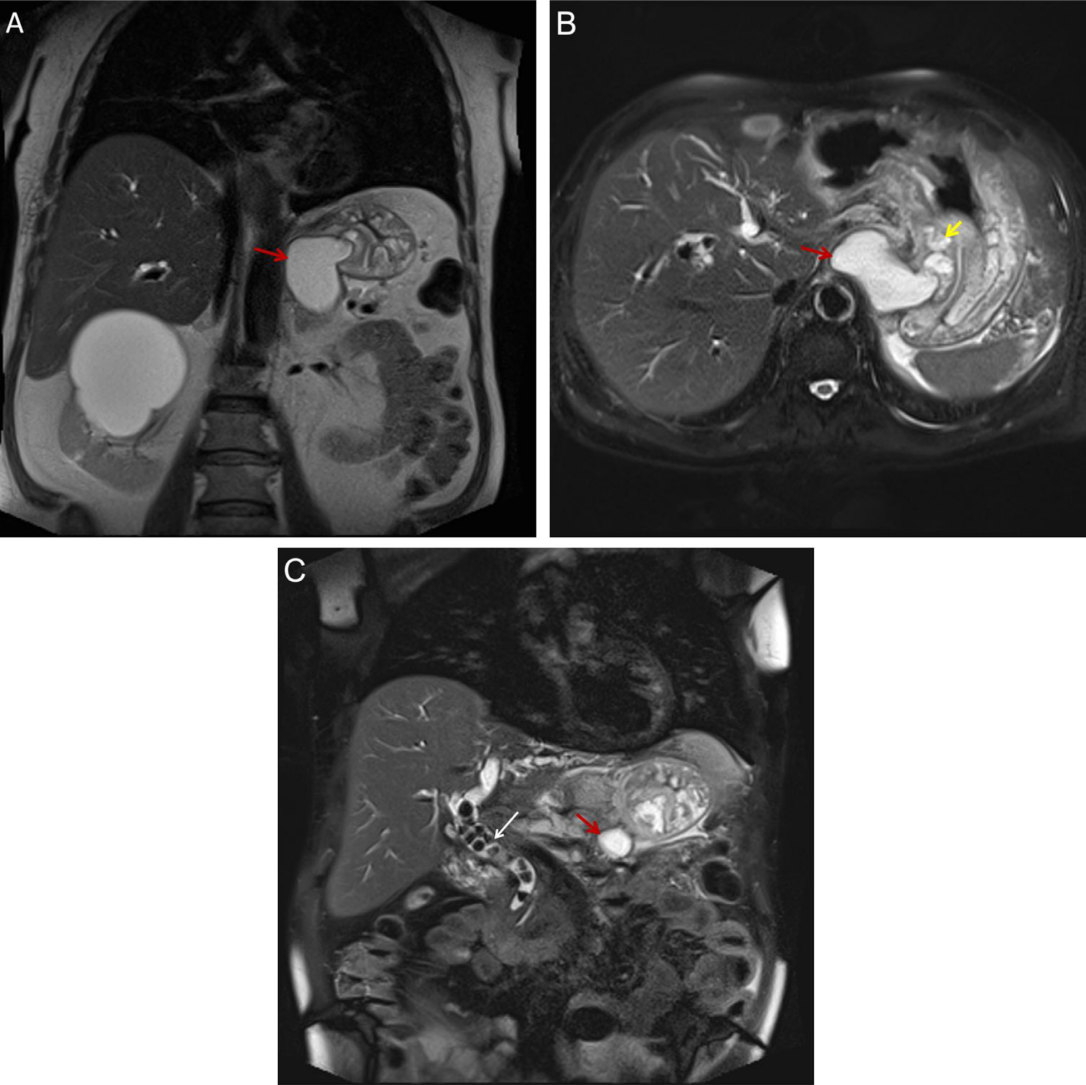
(A-C) Magnetic resonance cholangiopancreatography (MRCP), coronal and axial views: Evidence of a cystic structure with high T2 signal intensity arising from the lesser curvature of the stomach (red arrow). Additionally, there is evidence of multiple other cystic structures in the intramural aspect of the lesser curvature of the stomach (yellow arrow). Multiple stones are seen in the common bile duct, associated with dilation (white arrow) as a cause of recurrent pancreatitis.

Magnetic resonance cholangiopancreatography (MRCP) showed multiple stones in the hepatic ducts and common bile duct (CBD). We also observed irregular dilation of the main pancreatic duct (MPD) at the tail of the pancreas and atrophic changes in the head and tail of the pancreas. The body and fundus of the stomach displayed significant inflammation and wall thickening, and the lesser curvature revealed a 65 × 55 mm cystic structure posterior to the fundal part of the stomach (Fig. 4).

### Patient outcome

The patient underwent endoscopic retrograde cholangiopancreatography (ERCP) during his last admission. After becoming stable and symptom-free, he was discharged from the hospital. Since then, all subsequent follow-up appointments have been uneventful.

### Literature review

We performed a systematic literature search using the PubMed, Embase, Scopus, and Web of Science (all databases) databases. A search was done using the different combinations of “gastric pseudocyst”, “gastric intramural pseudocyst”, “intramural pseudocyst”, “gastric”, “stomach” and “pseudocyst” keywords. There were no limitations on the publication date, language, or type of article set at this stage. After combining the search results, we identified a total of 597 articles. The articles were imported into EndNote version 20.0, where duplicates were manually reviewed and deleted, resulting in 233 articles remaining for title and abstract screening. Two medical doctors independently performed the title and abstract screening. The inclusion criteria were articles in English, human studies, and studies explicitly discussing gastric intramural pseudocyst formation. We excluded articles written in languages other than English, animal studies, unrelated articles, studies mentioning gastric pseudocyst detection along with etiology of ectopic or heterotopic pancreas, and partially related articles that didn't explicitly mention intramural gastric pseudocyst detection. Title and abstract screening identified 19 articles that met the inclusion criteria. Full-text screening was performed by the same reviewers, and all 19 studies were included.

These consisted mainly of case reports, but to ensure comprehensiveness given the rarity of the condition, we also included patients reported in letters, conference abstracts, and posters, as well as 2 articles published in sections named “Image of the Month”. To avoid case duplication, we excluded 1 article that shared the same authorship with another included study.

We identified a total of 24 patients with intramural gastric pseudocysts from the 18 included articles [[1], [2], [3], [4], [5], [6], [7], [8], [9], [10], [11], [12], [13], [14], [15], [16], [17], [18]]. Including our reported case, the total number of patients analyzed was 25 (Table 1). We then extracted and analyzed the data.

**Table 1:** Characteristics of Gastric Pseudocysts cases reported in the literature including our case.

Study	Age	Gender	Alcohol consumption	Underlying cause	History of pancreatitis	Pancreatic pseudocyst	Signs and symptoms	Size	Amylase fluid content	Treatment
Ahuja et.al [6]	36	Female	Yes	Alcohol	Acute	No	Nausea, Abdominal pain (Upper), Vomiting (Bilious), Weight loss.	4.4 cm	18170 U/L	Fluoroscopy-guided drainage
Atiq et. al [7]	46	Male	Yes	Alcohol	Acute	Yes	Abdominal pain, Vomiting, Nausea	Not mentioned	Not mentioned	Aspiration through Surgery
Chiu et. al [8]	32	Male	Yes	Alcohol	Chronic	Yes	Epigastric pain	2 cm	6320 U/L	Endoscopic aspiration
Dong et. al [9]	45	Female	No	Not mentioned	Yes, but the type not mentioned	Yes	Abdominal pain	4.6 cm	Not mentioned	Conservative
Hatamori et. al [10]	67	Male	No	type 1 autoimmune pancreatitis (AIP)	type 1 autoimmune pancreatitis (AIP)	No	Enlargement of the pancreas	7 cm	22743 U/L	EUS-guided cyst drainage
Koop et. al [4]	41	Male	Yes	Alcohol	Acute	No	Abdominal pain (upper)	4 cm	39152 U/L	Surgery
Kumar et. al [11]	30	Male	Yes	Alcohol	Acute	No	Epigastric pain	2.5 cm	132600 IU/L	Not mentioned
Luigiano et. al [12]	66	Male	Yes	Alcohol	Chronic	No	Epigastric pain, Heartburn, Vomiting	3 cm	108570 U/L	Fine needle aspiration (FNA)
Milici et. al [13]	53	Male	No	Not mentioned	Not mentioned	Yes	Epigastric pain (Postprandial), Fever	5 cm	Not mentioned	Conservative
Oka et. al [2]	56	Male	Yes	Alcohol	Chronic	Yes	Epigastric pain, Fever, Nausea, Vomiting	Not mentioned	24250 U/L	Conservative
Radke et. al [3]	41	Male	Yes	Alcohol	Chronic	No	Epigastric pain (Radiating to back), Inability to gain weight	Not mentioned	7000 U/L	Laparotomy
Radke et. al [3]	43	Male	Yes	Alcohol	Chronic	No	Epigastric pain, Fever, Nausea, Vomiting, Weight loss	Not mentioned	Not mentioned	Laparotomy
Rana et. al [1]	54	Male	Yes	Alcohol	Acute	No	Abdominal pain	6 cm	Not mentioned	Conservative
Rana et. al [1]	24	Male	Yes	Alcohol	Chronic	No	Abdominal pain	2 cm	Not mentioned	Surgery
Rana et. al [1]	36	Male	Yes	Alcohol	Acute	No	Abdominal pain	4 cm	Not mentioned	Conservative
Salazar et. al [14]	38	Male	Yes	Alcohol	Not mentioned	Yes	Abdominal pain	Not mentioned	Not mentioned	Surgery
Søreide et. al [15]	56	Male	Yes	Alcohol	Yes, but the type not mentioned	Yes	Unspecific abdominal complaints	4.2 cm	Not mentioned	Conservative
Søreide et. al [15]	69	Male	Yes	Alcohol	Chronic	No	Abdominal pain, Gastrointestinal bleeding	Not mentioned	Not mentioned	Endoscopic pancreatic stenting
Søreide et. al [15]	73	Male	Yes	Alcohol	Yes, but the type not mentioned	No	Abdominal pain	Not mentioned	Not mentioned	Conservative
Suraweera et. al [16]	41	Male	Yes	Alcohol	Chronic	Yes	Emesis (Nonbloody, Nonbilious), Epigastric pain, Nausea	1.2 cm	Not mentioned	Conservative
Suraweera et. al [16]	63	Male	Yes	Alcohol	Chronic	Yes	Abdominal pain, Emesis (Nonbloody, Nonbilious), Nausea	Not mentioned	61500 U/L	EUS guided drainage
Vitello et. al [17]	20	Male	Yes	Alcohol	Acute	Yes	Nausea, Vomiting	Not mentioned	240000 U / L	Surgery
Wagholikar et. al [5]	37	Male	Yes	Alcohol	Acute	Yes	Vomiting (Non bilus, Postprandial)	Not mentioned	9742 U/L	US-guided percutaneous aspiration
Zahlan et. al [18]	68	Male	Yes	Alcohol	Not mentioned	Not mentioned	Epigastric pain, Weight loss, Nausea, Vomiting	Not mentioned	Not mentioned	Surgery
Our study	57	Male	No	Probably due to recurrent Biliary Obstruction	Chronic	Yes	Epigastric pain, Nausea, Vomiting (Post prandial, Nonbloody, Nonbilious)	6.5 cm	Not mentioned	Conservative

**Fig. 5:**
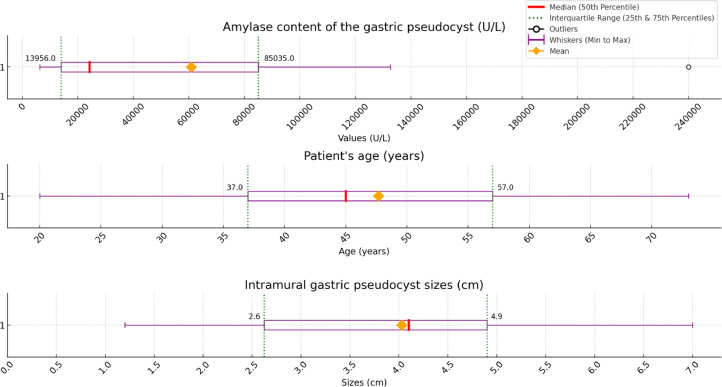
Characteristics of gastric pseudocysts reported in the literature including our case showing the median, mean, 25th percentile, 75th percentile, minimum, and maximum values.

Of the 25 patients, there were 23 males (92%) and 2 females (8%). The mean ± standard age of all patients was 47.68 ± 14.82, with a range of 20-73 years (Fig. 5). About 21 of the 25 patients (84%) had a history of abusive or chronic alcohol consumption. Three patients either did not mention pancreatitis, or we were unable to provide the necessary data. The remaining 22 patients confirmed positive for pancreatitis (88%): 10 had chronic pancreatitis, 7 had acute pancreatitis, 1 had Type 1 autoimmune pancreatitis (AIP), and 3 had no pancreatitis type mentioned. In 12 patients, the presence of a previous or current pancreatic pseudocyst was mentioned.

Common signs and symptoms were abdominal, especially epigastric pain (in 20 patients), vomiting or emesis (in 11 patients), nausea (in 9 patients), weight loss or inability to gain weight (in 4 patients), and fever (in 3 patients). Other symptoms were heartburn and gastrointestinal bleeding.

There were 14 patients with recorded intramural gastric pseudocyst sizes with a mean ± standard deviation of 4.02 ± 1.75 cm, ranging from 1.2 to 7.0 cm (Fig. 5). Measuring the amylase content of the gastric pseudocyst was done in 11 patients, with a mean ± standard deviation of 60,913.36 ± 72,862.76 U/L, ranging from 6320 U/L to 240,000 U/L (Fig. 5).

Laboratory data from the reported cases showed notable rises in total white blood cell count, serum amylase, serum lipase, and high C-reactive protein (CRP) levels.

Common treatment methods included conservative treatment (in 9 patients), different types of surgery (in 7 patients), and various nonsurgical methods of drainage using endoscopy, ultrasonography, fluoroscopy, and endoscopic ultrasound (in 9 patients).

One patient experienced a perforation into the stomach lumen and the formation of a hematoma. One patient passed away 6 months later in follow-up due to cardiac arrest (he had a comorbid medical history). The rest of the patients, for whom complications and outcomes were reported, had favorable and uneventful results.

### Pathophysiology

As previously mentioned, H. M. Radke and J. W. Bell came up with the term “intramural gastric pseudocyst” in 1966 [3]; Since then, similar definitions and pathophysiology for this condition have been proposed, but they all point to the pancreatic tissue and pancreatic pseudocyst [19].

Pancreatic pseudocysts are accumulations of pancreatic secretions enclosed by a fibrous layer, usually appearing following an episode of acute or chronic pancreatitis [[20], [21], [22]].

Pancreatitis can cause the pancreatic ducts to rupture, resulting in enzyme leakage [23]. These enzymes, in addition to necrotic tissue and inflammatory cells, accumulate in the pancreas or surrounding tissue. Over time, a fibrous tissue wall develops around this collection, resulting in the formation of a pseudocyst that lacks an epithelial lining. The process entails enzymes digesting inflamed tissue and forming a fibrous capsule [[24], [25], [26]]. The absence of an epithelial layer categorizes these cystic pancreatic lesions as nonepithelial cystic pancreatic lesions [20].

Pancreatic pseudocysts can result from a variety of causes, including alcohol consumption, biliary tract disorders, traumatic injuries, and surgical procedures. Among these, alcohol is the primary and most influential factor [24,27].

Pancreatic-related pseudocysts are typically found in the peripancreatic region, but they have also been sporadically observed in unusual locations such as the stomach, duodenum, spleen, liver, mediastinum, pelvis, and kidney [1,19].

The exact pathophysiology of gastric intramural pseudocysts remains unclear, but based on reported cases and suggestions from current studies, there are some possibilities. After a pancreatic pseudocyst forms, it usually resolves without requiring an invasive procedure. However, complications such as rupture and pancreatic fistulas can occur [26,28,29].

In cases of leakage, rupture, or fistula formation with nearby organs, viscera, cavities, or vessels, especially the stomach due to its anatomical proximity to the pancreas, the contents of a pancreatic pseudocyst, which include amylase, lipase, and other proteolytic enzymes, can cause erosion, inflammation, and extension into the gastric wall layers [26,[30], [31], [32], [33], [34], [35], [36]]. This can lead to perforation of the gastric walls, particularly in the presence of gastric ulcers, allowing the pseudocyst contents to drain spontaneously into the stomach cavity, resulting in hematoma, gastrointestinal bleeding, ischemia, or gastritis [[37], [38], [39], [40]].

Alternatively, the pseudocyst may not cause perforation and instead remain within the gastric wall layers. A fibrous tissue wall can form around this collection over time, which is similar to how pancreatic pseudocysts form. This speculative process can lead to the formation of an intramural gastric pseudocyst.

Another detected pathological way of forming an intramural gastric pseudocyst is through an ectopic pancreas. Ectopic, or heterotopic pancreas, is a congenital disorder identified by the presence of pancreatic tissue that is anatomically distinct from the main pancreas and has its ductal system. This condition is mostly seen in the upper gastrointestinal tract, including the stomach, duodenum, and jejunum [41,42]. Ectopic pancreatic tissue is prone to the same complications as the main pancreas, such as pseudocyst formation. Pseudocysts may develop in ectopic pancreatic tissue as a result of pancreatitis or duct obstruction. In cases of ectopic lesions and pancreatitis, pseudocyst formation is a frequent occurrence [[41], [42], [43], [44], [45]].

Gastric intramural pseudocysts related to ectopic pancreas are not the main focus of this study due to their different etiology and underlying causes. However, we mention them in various sections because they share common features with intramural gastric pseudocysts associated with the main pancreas.

### Diagnosis

Imaging modalities are instrumental in diagnosing intramural gastric pseudocysts. This condition can be diagnosed using ultrasonography (US), computed tomography (CT), magnetic resonance cholangiopancreatography (MRCP), and magnetic resonance imaging (MRI) [1,4,13,15,17].

Previous studies noted the use of the upper gastrointestinal series (UGI), which involves X-rays or fluoroscopy utilizing barium contrast [1,13,40].

To diagnose and understand the underlying cause of the disease more effectively, it is necessary to examine the pancreas using imaging techniques to identify the presence of pancreatitis (either chronic or acute), pancreatic pseudocysts, or any other complications in nearby organs. Contrast-enhanced computed tomography (CECT) is a valuable tool for confirming the presence of cystic lesions and identifying the characteristics of acute or chronic pancreatitis [1,4,5,12].

Upper gastrointestinal endoscopy and endoscopic ultrasound (EUS) are 2 other methods being used for evaluating intramural pseudocysts. EUS is considered the most reliable and effective modality for diagnosing and evaluating intramural gastric pseudocysts [1,36,46,47]. It is highly proficient at identifying the cyst contents and evaluating the different layers of the gastric wall. It is a very useful diagnostic tool for evaluating gastric submucosal tumors and cystic lesions. This method provides reliable information about the size, depth, internal features, and origin layer of these lesions [46,47].

Intramural gastric pseudocysts contain amylase-rich pancreatic fluid. To diagnose and confirm the origin of the disease, as well as to perform differential diagnosis, fluid aspiration from the cyst during EUS, endoscopy, or under ultrasonography guidance is utilized. The aspirated fluid is then examined for amylase levels; high amylase levels indicate a pancreatic source for the cystic lesion [1,5,8]. Additionally, carcinoembryonic antigen (CEA) levels are measured to distinguish between benign and malignant cystic lesions. Elevated CEA levels in the cyst fluid are more indicative of mucinous cystic neoplasms [[48], [49], [50]].

As a cystic structure, gastric intramural pseudocysts are similar to pancreatic pseudocysts in imaging studies. They appear as well-defined lesions arising from the gastric walls. Depending on the content of the cyst, which may include debris or necrotic tissue, the texture could be homogeneous or inhomogeneous on CT scans or may show internal echoes on ultrasound. The definite diagnosis of this condition is made through EUS and cyst fluid analysis, revealing high amylase content along with a correlation with the patient's medical history [16,51,52].

The differential diagnosis of intramural gastric pseudocysts involves differentiating them from other cystic lesions and masses in the stomach and adjacent structures. The most important differentials include pancreatic pseudocysts, mucinous cystic neoplasms, gastric submucosal tumors, duplication cysts, abscesses, gastrointestinal stromal tumors (GISTs), and leiomyomas [48,49,[53], [54], [55], [56], [57], [58], [59], [60]].

### Management and treatments

The management of intramural gastric pseudocysts could be divided into 3 main treatment strategies: conservative management, surgical interventions, and nonsurgical interventions [[1], [2], [3], [4], [5],^12^,^13^]. Conservative management entails administering analgesics to relieve pain, prescribing intravenous fluids to maintain the patient's hydration and electrolyte balance, and using antibiotics to treat or prevent infection. Nonsurgical interventions are usually less invasive procedures, and they often have lower risks and fewer possible complications. Suggested nonsurgical interventions are drainage of the cyst under the guidance of fluoroscopy, ultrasonography, endoscopic aspiration, and EUS-guided aspiration [5,6,8,10,22].

Physicians usually choose the treatment methods based on the underlying cause, complications, patients’ clinical status, vital signs, and their individual preferences.

In cases of severe or significantly symptomatic patients, or when a definite diagnosis is unclear or there are other underlying causes in the stomach, EUS-guided diagnosis and drainage could be the best treatment method [6,8,10]. However, in cases of more severe and potentially fatal conditions with other pathologies needing surgery, surgical interventions such as laparotomy (like our case) and gastrotomy may be required [3,53].

Procedure-related complications following drainage of pseudocysts or surgical interventions can occur, such as hemorrhage or bleeding, gastric perforation, or infection [[61], [62], [63]].

In reported cases of intramural gastric pseudocysts, most patients had favorable outcomes without any significant complications, regardless of the treatment method. However, more research is required to establish the best guidelines for treating this condition.

Additionally, in cases with a current episode of pancreatitis or complicated pancreatic pseudocysts, such as ruptures, peritonitis, gastric perforations, hematoma, or gastrointestinal bleeding, appropriate management of these complications is essential [6,7,9,10,14,16]. Furthermore, in the presence of ectopic pancreas, treatment strategies may differ, necessitating distinct strategies [42,44,45].

## Discussion

Only case reports and case studies have reported intramural gastric pseudocysts, making them extremely rare. Due to a lack of literature, it was important to discuss the pathophysiology, clinical presentation, patient profile, diagnostic methods, and treatment options of intramural gastric pseudocysts to effectively manage the disease and help with future research on it. To achieve this goal, we designed this case report and followed it with a literature review that provided an overview of reported cases.

Our reported case, a 57-year-old man with a history of chronic pancreatitis but no history of alcohol consumption, fits the common demographic identified in the literature. This deviation highlights the need for further research on intramural gastric pseudocysts, especially in populations with lower alcohol consumption.

Our case demonstrated an incidence of recurrence in the condition which is rarely seen in the available literature. This recurrence could be attributed to the patient's recurrent CBD stone formation or chronic pancreatitis. The patient's gastric pseudocysts were diagnosed in all imaging studies following the first surgery suggesting a possible link between the procedures performed on pancreatic pseudocysts and the recurrence. This observation warrants further evaluation and more research to understand the underlying mechanisms and potential contributing factors.

Our literature review and analysis of reported cases indicate that intramural gastric pseudocysts predominantly occur in middle-aged males (37–57 years) with a history of chronic or abusive alcohol consumption and pancreatitis.

Based on the suggested pathophysiology in the literature, there is a link between pancreatitis and pseudocyst formation in the pancreas with intramural gastric pseudocysts. Our analysis shows that nearly half of the patients had a history of diagnosed pancreatic pseudocysts.

The most common symptoms were abdominal pain, usually epigastric, nausea, vomiting, weight loss, and fever. These symptoms are not fully specific and can be seen in many gastrointestinal disorders.

Endoscopic ultrasonography (EUS) is the most effective method for diagnosing intramural gastric pseudocysts [36]. Endoscopic ultrasound (EUS) can be used to extract fluid from the cyst to examine the levels of amylase and carcinoembryonic antigen (CEA), which may help in differential diagnosis [48,49]. In addition, EUS offers comprehensive data on the lesion, making it an excellent tool for differentiating intramural gastric pseudocysts from other ailments. Additionally, it can serve therapeutic purposes like cyst drainage [46,47].

In our reported case, EUS and cyst fluid analysis were not performed because the cause of the patient's symptoms and referral to the hospital was believed to be recurrent biliary obstruction caused by CBD stones with the underlying cause of recurrent chronic pancreatitis.

As stated in Fig. 1, the patient's findings suggested acute necrotizing pancreatitis and pseudocyst formation simultaneously. Pancreatic pseudocysts usually mature and can be detected weeks after a necrotizing pancreatitis episode [21]. The patient's history of chronic pancreatitis could justify this coexistence. This episode could represent an acute-on-chronic pancreatitis episode.

There are several treatment options for intramural gastric pseudocysts, including surgery, nonsurgical interventions, and conservative management. Following conservative strategies (like our case) requires a comprehensive assessment of the patient's condition [1,5,13].

Different imaging modalities and radiologic features in computed tomography (CT), ultrasonography (US), and other modalities have been instrumental in diagnosing conditions like pancreatic and gastric pseudocysts for many years.

Our report demonstrated some of the disease's radiologic features and highlighted the diagnostic challenges associated with its rarity.

There is still a need for more research and study to better understand the pathophysiology of the disease and to provide more accurate management guidelines in the future.

## Conclusion

Intramural gastric pseudocyst is a very rare condition and typically can be seen in middle-aged males (37-57 years old) with a history of chronic or abusive alcohol use and pancreatitis.

More research is needed in the future to determine the pathophysiology and most effective treatment strategies for intramural gastric pseudocysts.

## Patient consent

We have obtained written informed consent from the patient to publish his case, including the use of medical history, clinical data, laboratory data, and images.
